# Protecting Important Sites for Biodiversity Contributes to Meeting Global Conservation Targets

**DOI:** 10.1371/journal.pone.0032529

**Published:** 2012-03-21

**Authors:** Stuart H. M. Butchart, Jörn P. W. Scharlemann, Mike I. Evans, Suhel Quader, Salvatore Aricò, Julius Arinaitwe, Mark Balman, Leon A. Bennun, Bastian Bertzky, Charles Besançon, Timothy M. Boucher, Thomas M. Brooks, Ian J. Burfield, Neil D. Burgess, Simba Chan, Rob P. Clay, Mike J. Crosby, Nicholas C. Davidson, Naamal De Silva, Christian Devenish, Guy C. L. Dutson, David F. Día z Fernández, Lincoln D. C. Fishpool, Claire Fitzgerald, Matt Foster, Melanie F. Heath, Marc Hockings, Michael Hoffmann, David Knox, Frank W. Larsen, John F. Lamoreux, Colby Loucks, Ian May, James Millett, Dominic Molloy, Paul Morling, Mike Parr, Taylor H. Ricketts, Nathalie Seddon, Benjamin Skolnik, Simon N. Stuart, Amy Upgren, Stephen Woodley

**Affiliations:** 1 BirdLife International, Cambridge, United Kingdom; 2 United Nations Environment Programme World Conservation Monitoring Centre, Cambridge, United Kingdom; 3 National Centre for Biological Sciences, Tata Institute of Fundamental Research, Bangalore, India; 4 United Nations Educational, Scientific and Cultural Organization, Paris, France; 5 BirdLife International Africa Partnership Secretariat, Nairobi, Kenya; 6 The Nature Conservancy, Arlington, Virginia, United States of America; 7 NatureServe, Arlington, Virginia, United States of America; 8 World Agroforestry Center, International Center for Research in Agroforestry, University of the Philippines, Los Baños, Philippines; 9 School of Geography and Environmental Studies, University of Tasmania, Hobart, Australia; 10 Center for Macroecology, Evolution and Climate, Department of Biology, University of Copenhagen, Copenhagen, Denmark; 11 Conservation Science Program, World Wildlife Fund, Washington, District of Columbia, United States of America; 12 BirdLife International Asia Regional Office, Tokyo, Japan; 13 BirdLife International Americas Secretariat, Quito, Ecuador; 14 Secretariat of the Ramsar Convention on Wetlands, Gland, Switzerland; 15 Conservation International, Arlington, Virginia, United States of America; 16 Birds Australia, Carlton, Australia; 17 Aves y Conservación, Quito, Ecuador; 18 National Fish and Wildlife Foundation, Washington, District of Columbia, United States of America; 19 School of Geography, Planning and Environmental Management, University of Queensland, Brisbane, Australia; 20 Species Survival Commission, International Union for Conservation of Nature, Gland, Switzerland; 21 The Wharton School, University of Pennsylvania, Philadelphia, Pennsylvania, United States of America; 22 BirdLife International Pacific Partnership Secretariat, Suva, Fiji; 23 Royal Society for the Protection of Birds, Sandy, United Kingdom; 24 American Bird Conservancy, Washington, District of Columbia, United States of America; 25 The Gund Institute for Ecological Economics, University of Vermont, Burlington, Vermont, United States of America; 26 Department of Zoology, Oxford, United Kingdom; 27 Department of Biology and Biochemistry, University of Bath, Bath, United Kingdom; 28 Al Ain Wildlife Park and Resort, Abu Dhabi, United Arab Emirates; 29 Natural Resources Branch, Parks Canada, Hull, Quebec, Canada; University of Kent, United Kingdom

## Abstract

Protected areas (PAs) are a cornerstone of conservation efforts and now cover nearly 13% of the world's land surface, with the world's governments committed to expand this to 17%. However, as biodiversity continues to decline, the effectiveness of PAs in reducing the extinction risk of species remains largely untested. We analyzed PA coverage and trends in species' extinction risk at globally significant sites for conserving birds (10,993 Important Bird Areas, IBAs) and highly threatened vertebrates and conifers (588 Alliance for Zero Extinction sites, AZEs) (referred to collectively hereafter as ‘important sites’). Species occurring in important sites with greater PA coverage experienced smaller increases in extinction risk over recent decades: the increase was half as large for bird species with>50% of the IBAs at which they occur completely covered by PAs, and a third lower for birds, mammals and amphibians restricted to protected AZEs (compared with unprotected or partially protected sites). Globally, half of the important sites for biodiversity conservation remain unprotected (49% of IBAs, 51% of AZEs). While PA coverage of important sites has increased over time, the proportion of PA area covering important sites, as opposed to less important land, has declined (by 0.45–1.14% annually since 1950 for IBAs and 0.79–1.49% annually for AZEs). Thus, while appropriately located PAs may slow the rate at which species are driven towards extinction, recent PA network expansion has under-represented important sites. We conclude that better targeted expansion of PA networks would help to improve biodiversity trends.

## Introduction

With biodiversity coming under increasing pressure and continuing to disappear [1], PAs are regarded as a core strategy for conserving nature [2]. Consequently, the 193 Parties to the Convention on Biological Diversity (CBD) recently adopted a target to conserve effectively 17% of terrestrial (and inland water) areas and 10% of coastal and marine areas by 2020, ‘especially areas of particular importance for biodiversity…’ [3]. Over 150,000 PAs – established and managed for long-term conservation of nature [4] – have been designated so far, covering 12.9% of the earth's terrestrial surface outside Antarctica [5].

Although PAs are often under considerable human pressure [2], [6], increasingly isolated [6], [7], under-resourced [8], ineffectively managed [9], and/or insufficient alone to achieve effective biodiversity conservation [10], it is often assumed that they help to reduce the loss, degradation and fragmentation of natural habitats and prevent declines and extinctions of threatened species. While there is reasonable evidence for PAs reducing rates of habitat loss [11]–[15], there is mixed evidence for their benefit in maintaining species' populations [16]–[20], and the effectiveness of PAs in reducing extinction risk remains largely untested.

PAs provide reasonable coverage of biodiversity at broad scales: half of 821 terrestrial ecoregions and eight of 14 biomes have ≥10% of their area protected [5], 14% of forest in 34 biodiversity hotspots is protected [21], and 88% of 11,633 vertebrate species (including 80% of 3,896 threatened vertebrates) have distributions that overlap with at least one PA [22], [23]. However, the global coverage of sites of particular importance for biodiversity, as called for in the CBD target [3], has hitherto not been quantified, nor have trends in this been evaluated.

We assessed trends in species' extinction risk (i.e. the aggregate rate at which species move towards extinction) and extent and trends in PA coverage for two subsets of ‘key biodiversity areas’ [24] (hereafter, ‘important sites’ for species conservation) with near-global coverage. IBAs are sites critical for the conservation of the world's birds; 10,993 such sites have been identified based on their populations of one or more of 4,445 threatened, restricted-range, biome-restricted or congregatory species [25] (see methods). AZEs hold ≥95% of the global population of any Critically Endangered or Endangered species, and hence are locations at which species extinctions are imminent unless appropriately safeguarded (i.e. protected or managed sustainably in ways consistent with the persistence of populations of target species) [26]; 588 such sites have been identified for 919 highly threatened vertebrate and conifer species.

**Figure 1:**
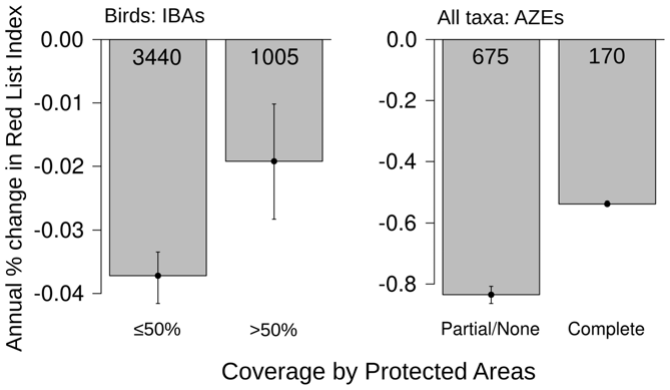
Annual percentage decline in Red List Index for sets of bird species (during 1988–2008) with ≤50% or >50% of IBAs completely protected, and for bird (1988–2008), mammal (1996–2008) and amphibian species (1980–2004) restricted to single sites (AZEs) that are partially/unprotected versus completely protected (averaged across taxa, weighting species equally). Numbers within each bar refer to the number of species. Error bars show 95% confidence intervals based on uncertainty around the estimated value that is introduced by Data Deficient species.

## Results

We investigated whether extent of protection was associated with differences in extinction risk trends of species occurring within important sites by examining two decades of Red List Index [27] trends (1988–2008) for 4,445 bird species of global conservation significance for which IBAs have been identified and for 845 birds, mammals and amphibians for which AZEs have been identified. The index measures aggregate extinction risk of sets of species and ranges from 1 (if none face imminent extinction) to 0 (if all are extinct). We found that the increase in extinction risk over the last two decades was half as large for bird species with >50% of the IBAs at which they occur completely covered by PAs (compared with species with ≤50% of IBAs completely covered; *P*<0.0001) and a third lower for birds, mammals and amphibians restricted to protected AZEs (compared with those restricted to unprotected or partially protected AZEs; *P*<0.0001) (Figs. 1, S1, S2). The observed trends for species differed significantly from those expected if protection of sites was assigned at random. Increases in extinction risk for species occurring in protected sites were significantly smaller than the distribution of values derived after randomly assigning species (in the observed proportions) as having >50% or ≤50% of IBAs protected, or completely protected vs incompletely/unprotected AZEs, and repeating this 10,000 times (Fig S3; *P*<0.05). It is unlikely that this was simply because less threatened sites may be more likely to be protected [28], [29], as the result for IBAs held even when excluding non-threatened species (annual % index decline = 0.186 for species with ≥50% of IBAs completely protected vs. 0.251 for species with <50% sites protected, *p* = 0.044), i.e. it cannot be explained by protected sites supporting non-threatened species and unprotected sites supporting threatened species. Furthermore, all AZEs are, by definition, under intense pressure (supporting the entire or overwhelming majority of the global population of at least one highly threatened species; [26]), yet we still found an association between degree of protection of these sites and reduction in RLI decline for species restricted to them. Finally, we found only a weak negative relationship between proportion of IBAs protected and local human population density (which is likely to be correlated with intensity of pressures) across all countries and levels of economic development (*F*_1,9114_ = 4.74, *P* = 0.03, slope±SE = −0.028±0.013), although this was stronger when the analysis was restricted to developing countries (*F*_1,4286_ = 14.25, *P* = 0.0002; slope±SE = −0.083±0.022).

**Figure 2:**
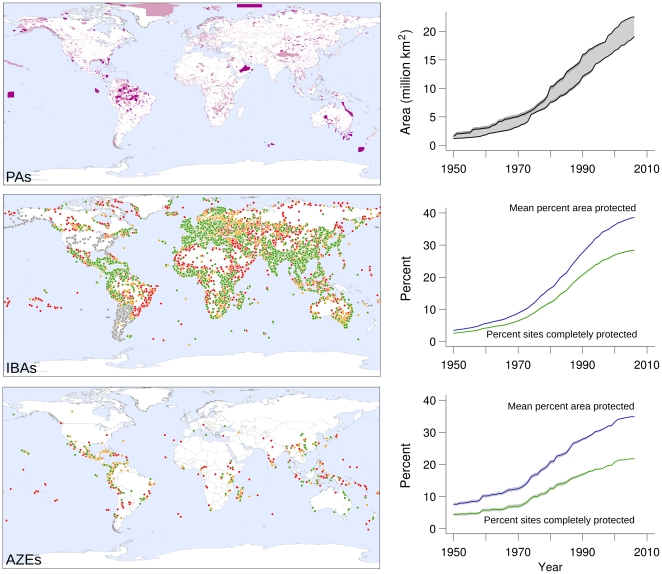
Distribution of PAs, IBAs, and AZEs showing (for the latter two) protected (green), partially protected (amber), and unprotected (red) sites, plus those of unknown protection status (grey), with trends in extent of PAs, and mean % area protected and % sites completely protected for IBAs and AZEs. Shading shows 95% confidence intervals based on uncertainty around date of protection (and, for a small subset of IBAs, proportion protected). For PAs, the lines represent minimum and maximum estimates with 95% confidence intervals, derived from PAs with delimited boundaries and PAs with and without delimited boundaries, respectively.

**Figure 3:**
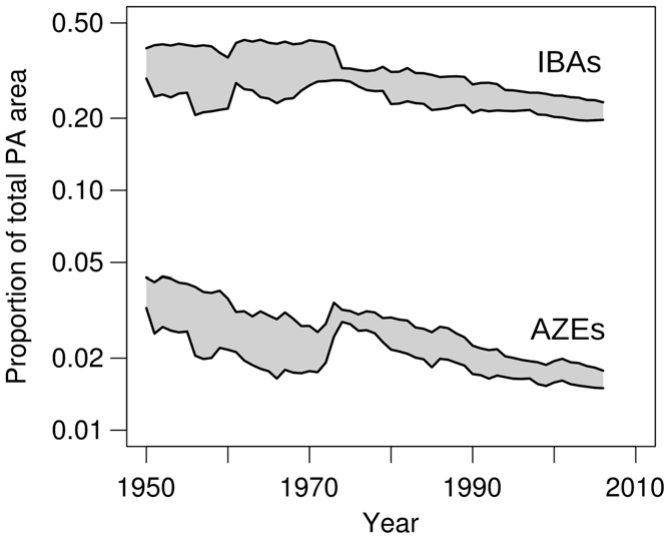
The proportion of total PA extent covering important sites, 1950–2006. Lines represent minimum and maximum estimates based on uncertainty in the extent of PAs.

Despite the association between protection of important sites and smaller inceases in extinction risk in target species, only 28% of IBAs are completely covered by PAs and 49% are wholly unprotected; on average only 39% of the area of each IBA is protected (73% excluding unprotected sites) (Fig. 2, Text S1). PA coverage of IBAs is lowest in the Brazilian Atlantic Forest, Middle East, northern Africa, freshwater ecosystems and deserts (Text S1; Table S1, Fig. S4). AZEs are marginally less well covered by PAs: 22% of sites are completely covered, 51% are unprotected; 35% of the area each site is covered on average (72% excluding unprotected sites) (Fig. 2, Text S1). Furthermore, the proportion of the total PA extent that covers important sites has declined significantly since 1950 for both IBAs (annual percent change between −0.45% and −1.14%; *P*<0.001, *N* = 57 years) and AZEs (−0.79 to −1.49%; *P*<0.001, *N* = 57 years); Fig. 3). PAs are therefore increasingly being designated outside important sites for species conservation, despite the high proportion of such sites that have yet to be protected.

## Discussion

### Using the Red List Index to assess biodiversity trends at important sites

The Red List Index is a useful approach for examining trends in the extinction risk of species, synthesising information on changes in species' population size, structure and trends and in their extent of distribution into a single index of aggregate survival probability (i.e. the inverse of extinction risk). As the system of Red List categories and criteria are designed to deal with uncertainty and paucity of information, they can be applied to all species globally within a taxonomic group, including poorly known tropical species (albeit with extremely poorly known species classified as Data Deficient). This allows comparisons to be made in the broad trends in extinction risk for different subsets of species globally. However, owing to the breadth of each Red List category, the Red List Index is only moderately sensitive. It is likely that many species in the taxonomic groups we considered experienced increases or decreases in extinction risk during the period, but insufficiently to cross thresholds for higher or lower Red List categories. Such trends are therefore not reflected in the index. Similarly, widespread abundant species that are classified as Least Concern may have declined in population size by up to 25% or may have increased substantially without such changes being reflected in revised Red List categories and hence incorporated into the Red List Index.

Assessing the impact of PAs on biodiversity trends would be best achieved through comparing population trends of target species within pairs of protected and unprotected sites that were matched to control for potentially confounding variables (size, location, human influence etc). However, population trend data representative of individual sites are extremely scarce for most taxa, even in the best-studied groups like large mammals and birds, and particularly in the tropics [30]. Given this constraint, we used a metric at the level of species (rather than of populations in individual sites), and of aggregate extinction risk across species (rather than population trends *per se*). It is reasonable to assume that adequate protection of the sole site harbouring the last remaining population of a species (in the case of AZEs) or of a suite of sites identified as the most important for the conservation of a species (IBAs) would affect the population trends, and habitat extent and condition, sufficiently to influence its International Union for Conservation of Nature (IUCN) Red List category of extinction risk. Hence, the Red List Index provides a useful tool for detecting moderately substantial differences in extinction risk trends for species occurring at sets of sites with different degrees of protection.

### Conserving important sites for biodiversity

IBAs and AZEs represent global networks of sites that are identified on the basis of current knowledge as the most important places for conserving biodiversity (specifically, threatened, restricted-range, biome-restricted or congregatory birds for the former and site-endemic threatened vertebrates and conifers for the latter [25], [26]). Effective conservation of all AZEs is by definition essential to achieve the CBD target of preventing extinctions of known threatened species [3] (all such sites are under threat and the loss of any one of them in the short- to medium-term would almost certainly result in global extinction of at least one species [26]), and it is highly likely that IBAs are the most urgent priorities for conservation action to achieve the CBD target of improving the status of known threatened species [3], at least for birds (and also to a significant degree for other taxa – see below). As the IBA identification process takes into account all available knowledge on the distribution of bird species, in theory no known important sites (as defined) for birds should be left outside the network, which indeed appears to be the case [31], although testing this using truly independent data is challenging, by definition. The IBA identification process often involves multiple stakeholders, considerable fieldwork and public outreach by local organizations, enhancing the effectiveness of protection and management through motivating local communities and relevant stakeholders [25].

However, recently designated PAs do not appear to have been well targeted towards these important but unprotected sites; this may have occurred for several interrelated reasons. PAs tend to be biased to higher elevations, steeper slopes, greater remoteness and lower suitability for agriculture [28], [29], rather than towards locations where they can best mitigate the rapid/extensive land-use change that threatens most species [25], [32]. Covariance between species richness/endemism and human population density [33] suggests that areas of highest biological value are typically more financially, socially and politically costly to protect. Human population density, while not the only determinant, is likely to be highly correlated with the logistical, political and financial cost of site protection. However, the proportion of IBAs protected was only weakly correlated with local human population density (although more strongly so in developing countries).

Other explanations for poor coverage of important sites by PAs could be that governments may lack awareness of, or be reticent to use, information on IBAs and AZEs in PA planning. Further, although the conservation importance and need for protection of many of these sites has been known for decades (indeed, many were already designated as PAs when identified as IBAs or AZEs), their documentation as such occurred relatively recently (since the 1980s for IBAs, and 2004 for AZEs). Nevertheless, in some countries IBA inventories have played an important role in informing recent PA designation (e.g. Madagascar, Philippines, European Union) [25] or PA site expansion (e.g. Nicaragua) [34]. Finally, PAs may have been targeted primarily at wilderness areas, abiotic (e.g. hydrological) processes or locations for recreation, tourism, hunting, scenery or cultural interest rather than biodiversity *per se* [29]. Data are currently unavailable to distinguish which of these explanations are the most important.

Our results are of importance beyond birds and highly threatened restricted-range vertebrates. In 12 countries in which globally important sites have also been systematically identified for mammals, amphibians and certain reptile, fish, plant and invertebrate clades (see methods), IBAs represent 71±5.4% (mean±SE) of the number and 80±5.4% of the area of important sites for all these taxa. As just 39% of the area of IBAs is protected on average, important sites for non-avian taxa are also likely to be poorly covered by PAs.

AZEs represent the most irreplaceable subset of important sites: the 414 highly threatened species at 298 unprotected AZEs will likely be part of the next wave of extinctions unless urgent action is taken [26]. Expansion of PA networks to cover all partially/unprotected AZEs (459) and IBAs (8,106) would add a further 4.6 million km^2^, increasing terrestrial coverage from 12.9% to 17.5%. This would meet the 17% coverage target for 2020 agreed by the world's governments in the new CBD strategic plan [3].

Recent analyses have highlighted the utility of a ‘return-on-investment’ approach for determining the most efficient set of sites, given a fixed budget, particular land-costs and specified biodiversity objectives [23], [35]–[36]. Such an approach could be used to identify the most efficient way to incorporate protection of important sites into PA networks within individual countries (the scale at which decisions are taken about precisely which areas to protect and manage for biodiversity). However, we do not attempt to set specific priorities for future resource allocation. Rather, our analysis is a retrospective one, revealing the coverage and impact of protected area establishment to date.

As well as expansion of the PA network (through enlargement of individual sites and/or addition of new ones), PAs need enhanced management in order to conserve biodiversity effectively in the long term, because many face intense pressures. For example, while completely protected IBAs are significantly less threatened than IBAs with incomplete or no protection, 47% still face ‘high’ or ‘very high’ threats (Fig. S5). We estimate, using a simple model [37], that adequately managing currently protected IBAs would cost US$11,500 million annually, of which c.US$8,900 million is required within high-income countries and only c.US$235 million in low-income countries (Table S2). Incorporating management of unprotected IBAs (but excluding acquisition and opportunity costs) increases this to an annual total of c.US$23,000 million, (c.US$17,700 million in high-income and c.US$400 million in low-income countries). These estimates are crude, ignoring fine-scale variation in costs for example, but by comparison, annual expenditure on PAs in the mid-1990s was estimated at c.US$6,000 million (88% of which was spent in developed countries), with an annual shortfall of US$2,300 million (40% of which was in developed countries) [38]. Our data therefore suggest that the shortfall to manage adequately an expanded set of PAs covering important sites for biodiversity globally may be substantially larger, but these costs are heavily skewed to developed countries.

There has been considerable progress towards meeting global PA targets, but this has not delivered adequate coverage of important sites for species conservation. The new CBD strategic plan calls for expanded PA coverage by 2020 to target especially areas of particular importance for biodiversity [3]. IBAs and AZEs represent existing, systematically identified global networks of relevant sites. Adequately protecting and managing them would enhance the contribution of PAs towards reducing biodiversity loss, contribute to multiple CBD targets [3], and respond to calls for greater protection of species distributions [19], [22], biomes and ecoregions [39]. We conclude that better targeted site-scale conservation would help to address the current mismatch between expanding PA coverage and declining species trends.

## Methods

### Red List Index

The Red List Index [27], [40] shows trends in the survival probability of sets of species, based on their categorisation of extinction risk on the IUCN Red List (http://www.iucnredlist.org). These categories, ranging from Least Concern through Near Threatened, Vulnerable, Endangered, Critically Endangered, Extinct in the Wild and Extinct, are assigned using standardised criteria with quantitative thresholds for population and range size, structure and trends. The index uses changes in categories between repeated assessments owing to genuine improvement or deterioration in status of a species (i.e. excluding category changes caused by revisions in knowledge, taxonomy or Red List criteria) [27], [40]. In practice, this is achieved through retrospectively correcting earlier categorisations using the most recent information and taxonomy from the IUCN Red List and BirdLife International databases (http://www.birdlife.org/datazone/species/index.html) to ensure that the same species are considered throughout and that only ‘genuine’ changes are included [27]. An RLI value at time *t* is calculated as
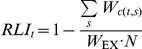



where *c*(*t,s*) is the IUCN Red List category of species *s* at time *t*, *W_c_* is the weight for category *c* (Extinct and Extinct in the Wild = 5,Critically Endangered = 4, Endangered = 3, Vulnerable = 2, Near threatened = 1, Least Concern = 0), *W*_EX_ is the weight assigned to extinct species; and *N* is the total number of assessed species, excluding those considered Data Deficient and those assessed as Extinct in the year the set of species was first assessed [40]. Red List categories are too broad for the Red List Index approach to reflect small or moderate changes in extinction risk over short time-frames for individual species, but the index is useful for examining overall trends for suites of species over multi-year time-scales [27], [40].

We calculated index trends firstly for 4,445 bird species of global conservation significance for which IBAs have been identified (1–542 IBAs per species, mean = 21.6±0.7), excluding one species classified as Extinct, three classified as Critically Endangered (Possibly Extinct) in 1988 [27] and 994 species from countries with incomplete IBA and/or PA coverage data (Table S3). We calculated indices for sets of species with ≤50% or >50% of IBAs completely covered by PAs by 2008 (the end point of each index). Second, we calculated indices for bird, mammal and amphibian species restricted to single sites (AZEs, 845 species, excluding 29 that were considered Critically Endangered (Possibly Extinct) in 1980) that have complete or partial/no PA coverage (by the end point of the index in each case: see below), weighting each species equally. We excluded reptiles (17 species), conifers (26) and corals (2), representing 4.9% of AZE species, from the index calculations, as trend data were unavailable. Different tests for IBAs and AZEs were necessary because all AZE species are, by definition, restricted to single sites, whereas most bird species ‘trigger’ multiple IBAs (i.e. for each species there are multiple sites that have sufficiently large populations to qualify under the criteria for IBA identification).

For both sets of Red List Indices we calculated the annual percentage decline in order to facilitate comparison between different taxonomic groups that were assessed over different time periods: 1988–2008 for birds, 1996–2008 for mammals, and 1980–2004 for amphibians. Error bars were calculated using a randomization procedure to reflect the uncertainty around the estimated index that is introduced by Data Deficient species [1]. We assumed that the true distribution of these species across Red List categories reflects the distribution of non-Data Deficient species, and randomly assigned each Data Deficient species to a Red List category with probability proportional to the distribution of non-Data Deficient species across categories. We did this for 23 bird species triggering IBAs and 14 species triggering AZEs (the latter assessed as Critically Endangered or Endangered when the AZE assessments were carried out, but as Data Deficient for the period for which RLI data were available). This random assignment was carried out 10,000 times, and the median, 2.5% percentile and 97.5% percentile of the resulting distribution of index values were taken as the central estimate and its lower and upper 95% limits respectively [1]. To test the significance of differences in annual percentage decline in Red List Indices, we calculated the difference between percent change in index values for each resample (for ≤50% versus >50% of IBAs completely covered by PAs, and for AZEs with complete versus partial/no PA coverage). We then asked whether (*P*>0.05) or not (*P*<0.05) zero was contained within the central 95% of these 10,000 resamples.

To test whether the observed annual percentage declines in Red List Indices for species occurring in protected IBAs and AZEs were significantly smaller than those expected by chance (and whether they were significantly larger than expected for those occurring in unprotected IBAs and AZEs), we randomly assigned species in the observed proportions as having ≤50% versus >50% of IBAs completely covered by PAs, or for AZEs with complete versus partial/no PA coverage, and repeated this 10,000 times (each with 1,000 resamples to assess uncertainty due to Data Deficient species, as above) (Fig. S3). We then asked whether (*P*>0.05) or not (*P*<0.05) the observed annual percentage declines in Red List Index were contained within the one-tailed 95% of these 10,000 resamples.

### IBAs and AZEs

‘Key biodiversity areas’ are important sites for species conservation, identified using quantitative criteria based on the presence of species for which site-scale conservation is appropriate: (a) globally threatened species, (b) restricted-range species, (c) congregations of species that concentrate at particular sites during some stage in their life cycle, and (d) biome-restricted species assemblages [24]. These four categories relate to threat (a) and irreplaceability (b–d), the two main considerations used in planning networks of sites for biodiversity conservation [41]. While such sites have been identified, at least in parts of the world, for birds (IBAs), plants, butterflies, mammals, certain freshwater taxa, and highly threatened taxa in certain groups (AZEs), only IBAs and AZEs have been identified across virtually all countries.

IBAs are places of international significance for the conservation of birds. They are identified (usually at a national scale through multi-stakeholder processes) using a standardised set of data-driven criteria and thresholds within the four categories listed above, ensuring that the approach can be used consistently worldwide [34], [42]–[45]. IBAs are delimited so that, as far as possible, they: (a) are different in character, habitat or ornithological importance from surrounding areas; (b) provide the requirements of the trigger species (i.e. those for which the site qualifies) while present, alone or in combination with networks of other sites; and (c) are or can be managed in some way for conservation. IBAs have been identified in almost all countries of the world [34], [42]–[45] but for the analyses presented here, we extracted data for 218 countries/territories from the World Biodiversity Database (WBDB; http://www.birdlife.org/datazone/sites/index.html), excluding data for 21 countries for which the dataset was incomplete (Table S3). We compared the overlap between IBAs and important sites identified for mammals, amphibians and certain reptile, fish, plant and invertebrate clades for 12 countries with available data: Bhutan, Cambodia, Ghana, Guinea, Laos, Liberia, Madagascar, Myanmar, Nepal, Philippines, Thailand and Vietnam using data from the WBDB.

AZEs are sites meeting three criteria: endangerment (supporting at least one Endangered or Critically Endangered species, as listed on the IUCN Red List); irreplaceability (holding the sole or overwhelmingly significant (≥95%) known population of the target species, for at least one life history segment); discreteness (having a definable boundary within which the character of habitats, biological communities, and/or management issues have more in common with each other than they do with those in adjacent areas) [26]. Terrestrial AZE sites have been identified globally for all mammals, birds, amphibians, selected reptile clades (Testudines, Crocodylia and Iguanidae) and conifers [26]. Our analyses were based on the 2010 AZE dataset (http://www.zeroextinction.org/search.cfm).

### PAs

The World Database on Protected Areas (WDPA; http://www.wdpa.org/) is the most comprehensive global spatial dataset on marine and terrestrial protected areas available. The WDPA is a joint project of the United Nations Environment Programme (UNEP) and IUCN, maintained at the UNEP World Conservation Monitoring Centre working with the IUCN World Commission on Protected Areas, governments and collaborating non-governmental organisations. To examine growth in extent of PAs, we used all nationally designated PAs (in IUCN Categories I–VI plus those without a category assigned), excluding internationally designated PAs and all sites with a status other than ‘designated’ [5]. We calculated a minimum PA network area by using 97,913 PAs for which a boundary polygon was available. We dissolved PA boundaries by country to remove overlaps using ArcGIS, taking the earliest designation year in such cases, and calculated PA coverage in Mollweide projection from the resulting 316,716 PA polygons. The maximum extent of PAs was calculated by using all PAs including those without a boundary polygon but which have an estimate of extent (35,350 PAs), assuming there is no overlap among such PAs or between them and those with boundary polygons. For analysing PA coverage of important sites, we overlaid PAs onto IBAs and AZEs, and updated the results where appropriate with current data from BirdLife and AZE partners on site protection. For 151,773 dissolved PA polygons, 55 IBAs and 27 AZEs with unknown PA establishment date, and 543 IBAs known (from national experts) to be partially protected but to an unknown extent, we randomly assigned a date or proportion protected from another site in that country, or where <2 sites with known date/proportion protected occurred in the country we randomly selected from all sites, repeating this procedure 1,000 times, plotting the mean and 95% confidence intervals.

We present data on AZEs and IBAs separately, but note that 50% of AZE sites also qualify as IBAs (representing 2.5% of IBAs) because they are triggered either by an AZE bird species or by a wider-ranging bird species that co-occurs with an AZE non-bird species. For analyses combining both AZE and IBA data, these sites counted only once.

### Population density around IBAs

We calculated mean human population density in IBAs and within 50 km buffers of them by overlaying buffered IBA polygons onto the CIESIN gridded human population density dataset [46] using the Geospatial Modelling Environment [47]. We ran a general linear mixed model with restricted maximum likelihood estimation on 9,281 IBAs with data, and entered population density (log transformed) as the dependent variable, proportion of area protected as a continuous fixed effect, developmental status (developed, developing and CIS) as a categorical fixed effect, and country as the random effect. Development status of a country was included as this factor has a strong effect on mean population density.

### Threats to IBAs

We took data on magnitude of threats to IBAs [48] from the WBDB for 2,000 IBAs in 101 countries for which standardized data were available. Threats to IBAs are scored by national IBA coordinators based on information collected at each IBA by site-based monitors, along with any other available information. The timing, geographic scope (% of population of ‘trigger’ species for which the site is identified, or % area) and severity (rate of decline in trigger species population or deterioration in area) for each threat type [49] is scored for each IBA (on a scale of 0 to 3), and the threat impact is calculated from these parameters [48]. Depending on the information available, each threat may be assessed based on its effect on all trigger species collectively, or on each individually, with the highest impact score for any species being used following a ‘weakest link’ approach. Thus the highest impact score of any threat determines the overall threat score for the IBA, following the same ‘weakest link’ approach [48].

### Management costs

We used a model [37] to estimate annual management costs of IBAs. It uses the relationship between the cost of site management per km^2^ and explanatory variables including PA size and Gross Domestic Product (GDP), Gross National Income (GNI), Purchasing Power Parity (PPP) and area of each country to assess recurrent management costs for effective field-based conservation. It therefore omits costs of land acquisition, compensation or any other fixed one-off expenditure. We applied this approach to IBAs (analysing the area protected and unprotected separately), using data on GDP and GNI [50], PPP [51] and country area [52]. PPP data were not available for 78 countries (with 8.6% of the total IBA area), so the final costs for countries in different income categories (low, lower-middle, upper-middle and high; [53] were scaled up uniformly by 8.6% to give total costs of managing all IBAs. Estimates in 2000 US$ from the model were converted to 2009 US$ using GDP deflator figures [54]. While estimates for individual sites may be unreliable, errors are likely to balance out for the gross estimate of annual management costs across the entire IBA network of >10,000 sites. Our approach assumes that each IBA would be managed as a single PA, which is reasonable given that these sites are identified as actual or potential management units.

## Supporting Information

Text S1 **Coverage of IBAs and AZEs by PAs and by internationally designated sites, and site-scale conservation under climate change.**
(DOC)

Figure S1 **Red List Index of species survival for species triggering IBAs of which over 50% are completely protected, compared with those for which≤50% are completely protected.** Shading indicates the 95% confidence intervals based on uncertainty around the estimated value that is introduced by Data Deficient species.
(TIF)

Figure S2 **Annual percentage decline in Red List Index for bird species (during 1988–2008) with different proportions of IBAs completely protected.** Numbers within each bar refer to the number of species. Error bars show 95% confidence intervals based on uncertainty around the estimated value that is introduced by Data Deficient species.
(TIF)

Figure S3 Observed annual percentage declines in Red List Index (RLI) are significantly different from those expected by chance based on 10,000 randomisations for (A) bird species (during 1988–2008) with>50% of IBAs completely protected (*N* = 1,004, *P*<0.001), and (B) for bird (1988–2008), mammal (1996–2008) and amphibian species (1980–2004) restricted to single sites (AZEs) that are partially/unprotected (*N* = 675, *P* = 0.025) versus completely covered by PAs (*N* = 170, *P* = 0.032). The RLI for bird species with≤50% of IBAs completely protected was not significantly different from random (*N* = 3440, *P* = 0.31; A). The observed annual percentage change in RLI is shown as red lines (with 95% confidence intervals based on uncertainty introduced by Data Deficient species shown by dashed lines, as in Fig. S1), and annual percentage change in RLI from randomly allocating species 10,000 times is shown by gray bars, with black lines indicating the 5% confidence interval for a one-tailed test.
(TIF)

Figure S4 Trends in mean % area protected for IBAs in different (A) habitats and (B) regions. Shading shows 95% confidence intervals based on uncertainty around date of protection (and, for a small subset of IBAs, proportion protected).
(TIF)

Figure S5 **Completely protected IBAs (n = 737) are significantly less threatened than partially/unprotected IBAs (n = 1,263; chi-squared test: χ^2^ = 19.0, df = 3,** ***P*** **<0.001), but almost half (47%) face ‘high’ or ‘very high’ threats.**
(TIF)

Table S1 **PA coverage (% area) for IBAs in different ecosystems, habitats, regions, and relevant to different Multilateral Environmental Agreements.**
(DOCX)

Table S2 **Costs of IBA management.**
(DOCX)

Table S3 **List of countries excluded from the analysis of PA coverage of IBAs owing to incomplete data on IBAs and/or their PA coverage.**
(DOCX)
