# Supplementary material for: Protecting Important Sites for Biodiversity Contributes to Meeting Global Conservation Targets
Source: PLoS One. 2012 Mar 21;7(3):e32529. doi: 10.1371/journal.pone.0032529 (PMC3310057; doi:10.1371/journal.pone.0032529)
Supplement: Text S1 — Coverage of IBAs and AZEs by PAs and by internationally designated sites, and site-scale conservation under climate change. (DOC) [file pone.0032529.s001.doc]

**Text S1**

*Coverage of IBAs and AZEs by PAs*

The number of dated, nationally designated PAs (with polygon dissolved/without a polygon included in the WDPA) increased from 10,338/1,794 polygons/sites covering 0.84/0.25 million km^2^ in 1950 to 178,790/21,169 polygons/sites covering 14.94/2.31 million km^2^ in 2006. The totals for 2010 (179,096/21,197 polygons/sites covering 15.19/2.32 million km^2^) are likely underestimates owing to time-lags in data acquisition. Including undated sites (with polygon dissolved/without a polygon) and assuming these were designated pre-1950, the number increased from 147,958/15,947 polygons/sites covering 4.95/1.21 million km^2^ in 1950 to 316,410/35,322 covering 19.05/3.27 million km^2^ in 2006 (and 316,716/35,350 covering 19.30/3.28 million km^2^ in 2010). By 2010, 38.0% of IBAs were more than 50% covered by PAs and 28.7% were more than 90% covered. For AZEs, 34.9% were more than 50% covered and 26.0% were more than 90% covered.

PA coverage of IBAs varied regionally (Table S1, Fig. S4). The mean % area of IBAs covered was higher in developed (43%) than developing (39%) countries, highest in Australasia (47.1%) and Asia (44.7%) and lowest in Middle East (18.6%) and Oceania (8.6%, although identification of IBAs is still underway in some countries this region). Coverage was higher in terrestrial ecosystems (43%) than freshwater (40%), highest in forest (47%) and grassland (43%) and lowest in desert (30%). Of sites relevant to each of three multilateral environmental agreements, PA coverage was greatest for the Agreement on the Conservation of Albatrosses and Petrels (46%), intermediate for the Ramsar Convention on Wetlands (41%) and lowest for the African-Eurasian Waterbird Agreement (36%).

A degree of overlap between PAs and IBAs or AZEs is unsurprising, since sites in both of the latter networks are identified as actual or potential management units. Nevertheless, precise boundary definitions for IBAs and AZEs take into account the distribution of habitat and populations of the species triggering site identification, and this partly explains why 28% of IBAs (3093) and 27.4% of AZEs (161) are only partially protected.

*Coverage of IBAs and AZEs by internationally designated sites*

We also examined coverage of IBAs and AZEs by two networks of internationally designated sites. The World Heritage Convention recognises cultural and natural sites of ‘Outstanding Universal Value’ including those that ‘contain the most important and significant natural habitats for in-situ conservation of biological diversity’ (criterion x) and those that are ‘outstanding examples representing significant on-going ecological and biological processes in the evolution and development of terrestrial, fresh water, coastal and marine ecosystems and communities of plants and animals’ (criterion ix). While not all IBAs and AZEs may meet the convention’s required conditions of intactness and effective management, these site networks clearly include important candidates for World Heritage designation. However, only 1.7% of IBAs (185) and 6% of AZEs (33) have been designated as World Heritage sites to date.

IBAs in Europe, the Middle East, Africa and Asia have been assessed for whether they meet the thresholds for criteria for designation as Wetlands of International Importance (‘Ramsar sites’) through the Ramsar Convention (1-4). These criteria include thresholds for total numbers of waterbirds and minimum numbers for individual species. Of 4,165 IBAs meeting these thresholds, 17.1% (711) have been designated (at least in part) as Ramsar sites to date, ranging from 7.9% in Asia to 24.4% in Europe.

Clearly, there are a large number of IBAs and AZEs of apparently international significance that have yet to be recognised through the relevant multilateral conventions. It seems likely that designation of such sites confers added protection and benefits for the biodiversity within them. For example, Ramsar designation of sites in Africa and North America has reportedly helped to reduce threats through increased public awareness and support for site-protection, improved local stakeholder participation in management, increased access to domestic and international conservation funding, and enhanced opportunities for promoting both scientific research and ecotourism (5,6). Further critical analyses are needed to quantify the benefits of Ramsar designation.

*Site-scale conservation under climate change*

Expansion of PA networks will also require consideration of the impacts of climate change and the associated shifts in the ranges of many species triggering IBA or AZE identification, including beyond the boundaries of some of the sites they currently occur in. However, recent analyses show that for IBAs at least, the network as a whole will continue to provide reasonable coverage of suitable climatic conditions for a high proportion of species (7), although the network may be less effective under more extreme scenarios (8). Hence, protecting these sites as components of regional and global networks will be essential for mitigating the worst impacts of climate change on biodiversity (7,9) in combination with their adaptive management, expansion, improved connectivity and wider landscape conservation (10). As many such sites are forested, their conservation would also help to minimize emissions of greenhouse gases from deforestation and forest degradation.

While site-scale conservation is an urgent priority, safeguarding biodiversity also requires complementary approaches at the landscape-scale and, in some cases, *ex situ*. For example, 18% of threatened mammals, birds, turtles and amphibians require broad-scale action in the short to medium term (11), mostly in addition to interventions at the site scale.

1. Evans MI, editor (1994) Important Bird Areas in the Middle East. Cambridge: BirdLife International. 410 p.
2. BirdLife International (2001) Important Bird Areas and Potential Ramsar Sites in Europe. Cambridge: BirdLife International. 126 p.
3. BirdLife International (2002) Important Bird Areas and Potential Ramsar Sites in Africa. Cambridge: BirdLife International. 148 p.
4. BirdLife International (2005) Important Bird Areas and Potential Ramsar Sites in Asia. Cambridge: BirdLife International. 96 p.
5. Gardner RC, Connolly KD (2007) The Ramsar Convention on Wetlands: Assessment of International Designations within the United States. Env Law Rev 37: 10089-10113.
6. Gardner RC, Connolly KD, Bamba A (2009) African Wetlands of International Importance: assessment of benefits associated with designations under the Ramsar Convention. Georgetown Int Env Law Rev 21: 257-294.
7. Hole DG, Huntley B , Pain DJ, Fishpool LDC, Butchart SHM, et al. (2009) Projected impacts of climate change on a continent-wide protected area network. Ecol Lett 12: 420–431.
8. Coetzee BWT, Robertson MP, Erasmus BFN, van Rensburg BJ, Thuiller W (2009) Ensemble models predict Important Bird Areas in southern Africa will become less effective for conserving endemic birds under climate change. Global Ecol Biogeogr 18: 701–710.
9. Hannah L (2010) A global conservation system for climate-change adaptation. Conserv Biol 24: 70–77.
10. Hole DG, Huntley B, Collingham YC, Fishpool LDC, Pain DJ et al. (2011) Towards a management framework for protected area networks in the face of climate change. Conserv Biol 25: 305–315.
11. Boyd C, Brooks TM, Butchart SHM, da Fonseca GAB, Hawkins AFA, et al. (2008) Spatial scale and the conservation of threatened species. Conserv Lett 1: 37–43.
