# Supplementary figures and images for: Protecting Important Sites for Biodiversity Contributes to Meeting Global Conservation Targets
Source: PLoS One. 2012 Mar 21;7(3):e32529. doi: 10.1371/journal.pone.0032529 (PMC3310057; doi:10.1371/journal.pone.0032529)

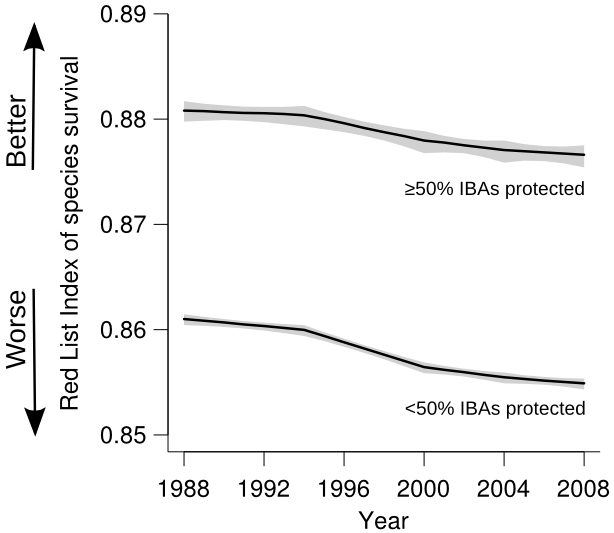

Supplement: Figure S1 — Red List Index of species survival for species triggering IBAs of which over 50% are completely protected, compared with those for which≤50% are completely protected. Shading indicates the 95% confidence intervals based on uncertainty around the estimated value that is introduced by Data Deficient species. (TIF) [file pone.0032529.s002.tif]

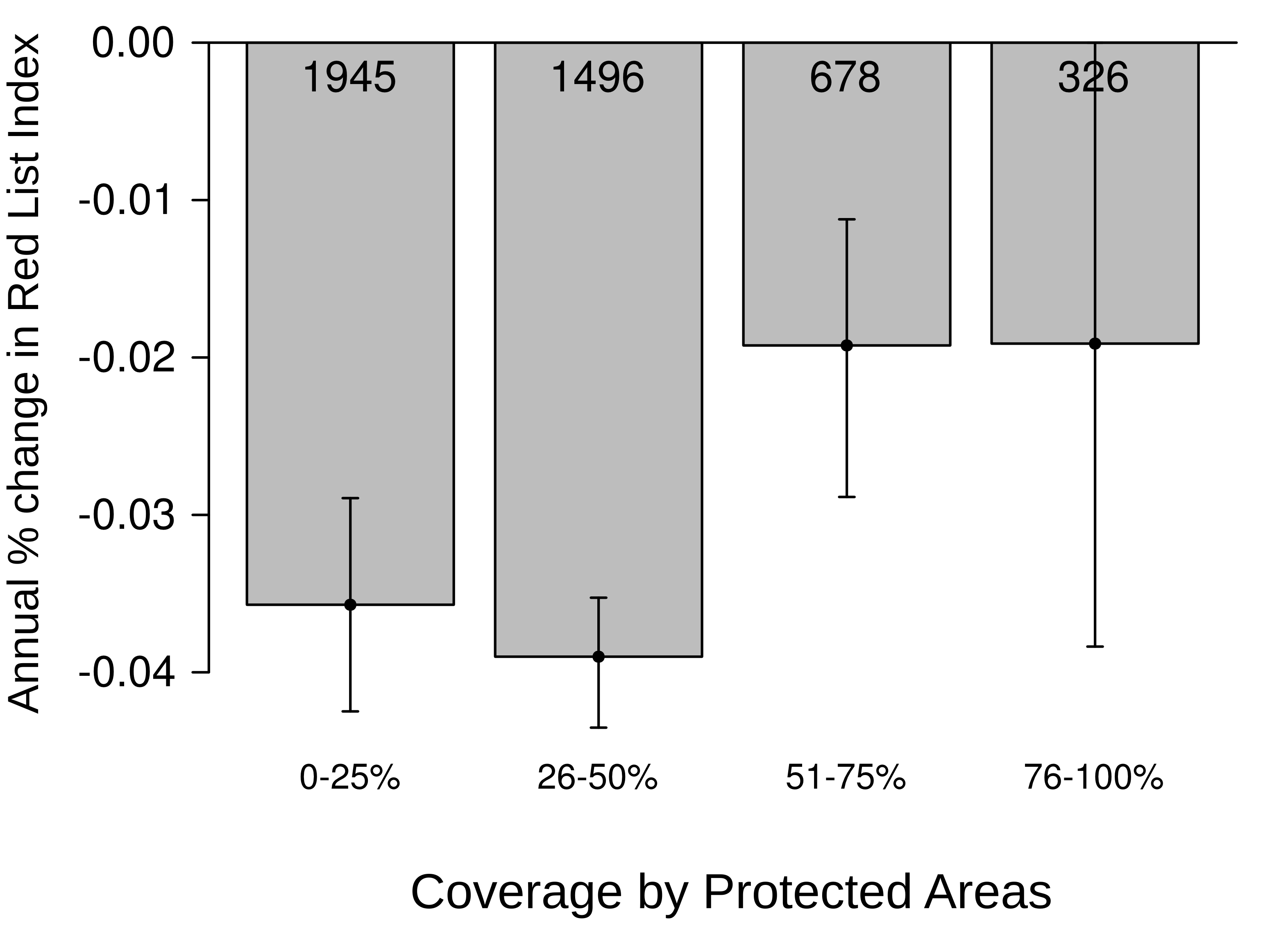

Supplement: Figure S2 — Annual percentage decline in Red List Index for bird species (during 1988–2008) with different proportions of IBAs completely protected. Numbers within each bar refer to the number of species. Error bars show 95% confidence intervals based on uncertainty around the estimated value that is introduced by Data Deficient species. (TIF) [file pone.0032529.s003.tif]

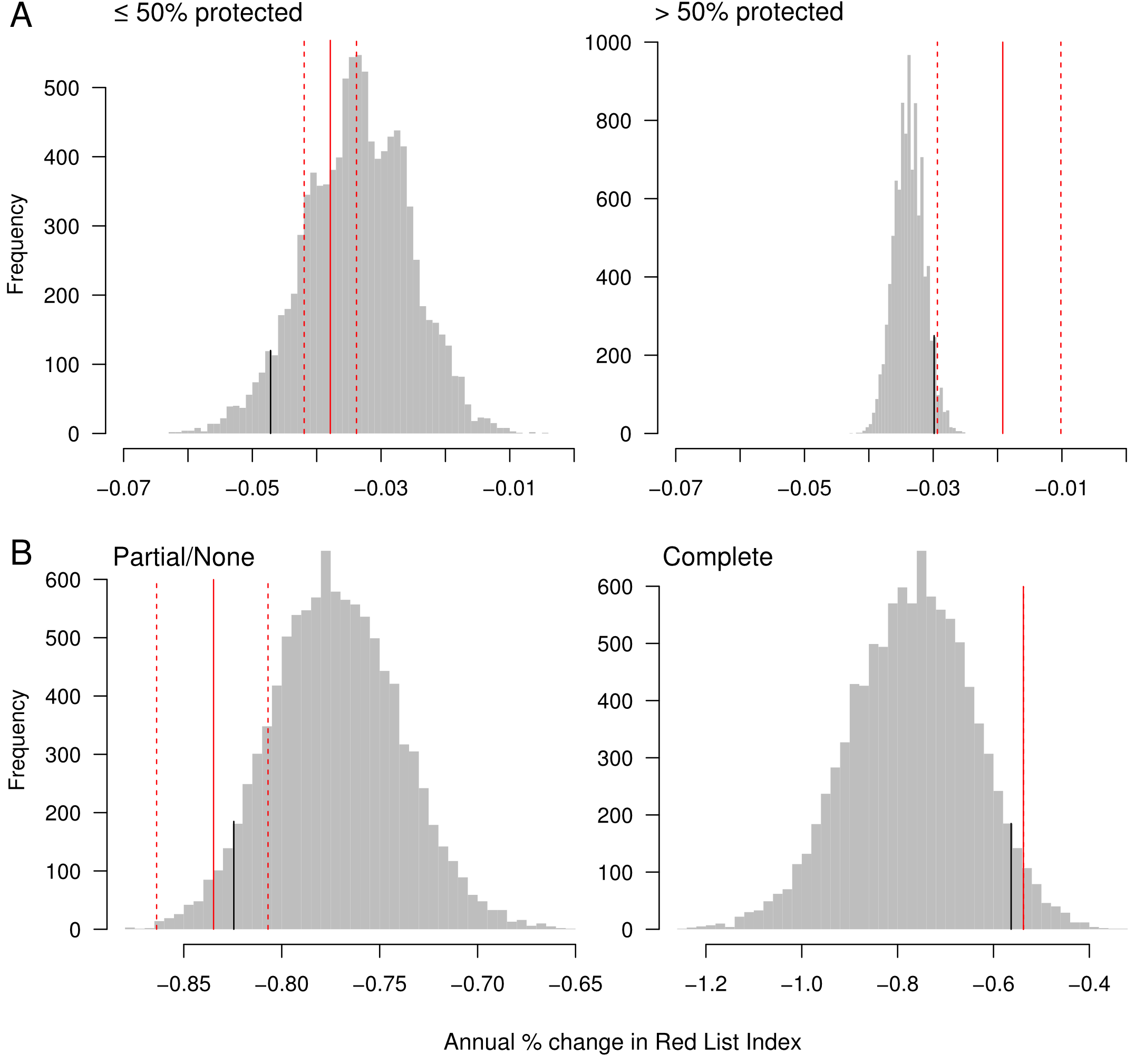

Supplement: Figure S3 — Observed annual percentage declines in Red List Index (RLI) are significantly different from those expected by chance based on 10,000 randomisations for (A) bird species (during 1988–2008) with>50% of IBAs completely protected (N = 1,004, P<0.001), and (B) for bird (1988–2008), mammal (1996–2008) and amphibian species (1980–2004) restricted to single sites (AZEs) that are partially/unprotected (N = 675, P = 0.025) versus completely covered by PAs (N = 170, P = 0.032). The RLI for bird species with≤50% of IBAs completely protected was not significantly different from random (N = 3440, P = 0.31; A). The observed annual percentage change in RLI is shown as red lines (with 95% confidence intervals based on uncertainty introduced by Data Deficient species shown by dashed lines, as in Fig. S1), and annual percentage change in RLI from randomly allocating species 10,000 times is shown by gray bars, with black lines indicating the 5% confidence interval for a one-tailed test. (TIF) [file pone.0032529.s004.tif]

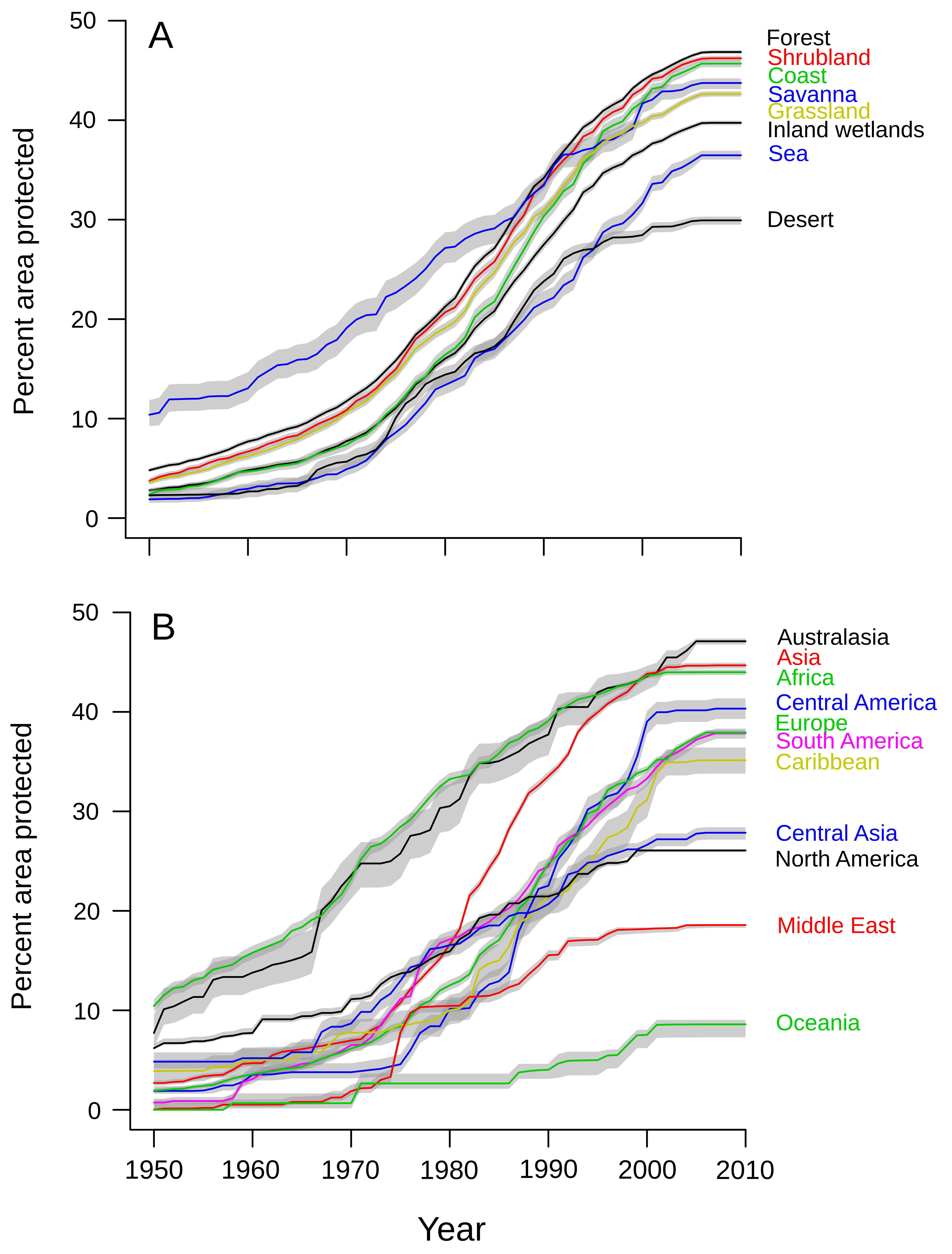

Supplement: Figure S4 — Trends in mean % area protected for IBAs in different (A) habitats and (B) regions. Shading shows 95% confidence intervals based on uncertainty around date of protection (and, for a small subset of IBAs, proportion protected). (TIF) [file pone.0032529.s005.tif]

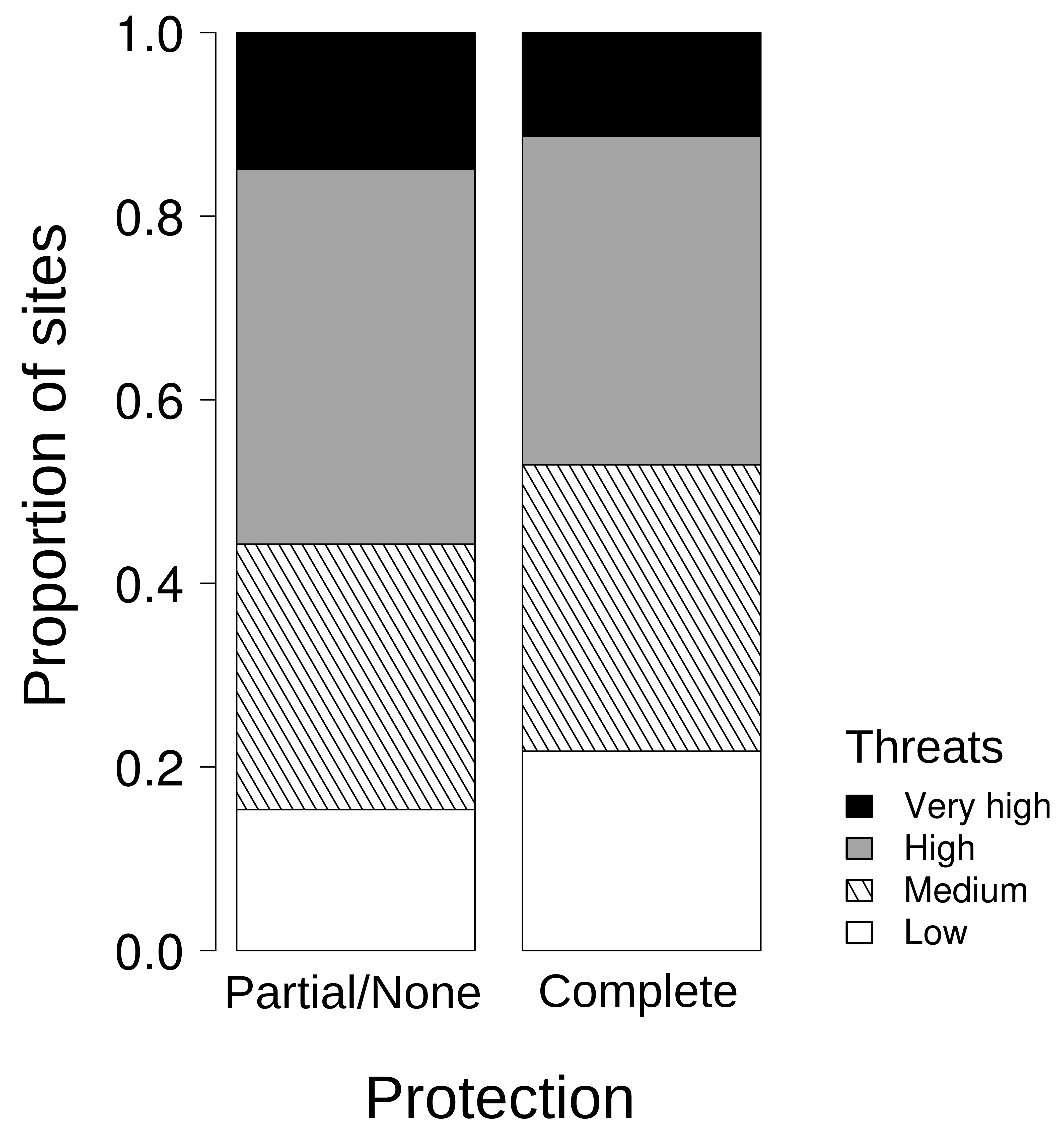

Supplement: Figure S5 — Completely protected IBAs (n = 737) are significantly less threatened than partially/unprotected IBAs (n = 1,263; chi-squared test: χ2 = 19.0, df = 3, P <0.001), but almost half (47%) face ‘high’ or ‘very high’ threats. (TIF) [file pone.0032529.s006.tif]
