# Supplementary material for: Protecting Important Sites for Biodiversity Contributes to Meeting Global Conservation Targets
Source: PLoS One. 2012 Mar 21;7(3):e32529. doi: 10.1371/journal.pone.0032529 (PMC3310057; doi:10.1371/journal.pone.0032529)
Supplement: Table S1 — PA coverage (% area) for IBAs in different ecosystems, habitats, regions, and relevant to different Multilateral Environmental Agreements. (DOCX) [file pone.0032529.s007.docx]

**Table S1.** PA coverage (% area) for IBAs in different ecosystems, habitats, regions, and relevant to different Multilateral Environmental Agreements.

|  | **Year** | | | |  |
| --- | --- | --- | --- | --- | --- |
|  | **1950** | **1990** | **2000** | **2009** | **No. sites** |
| **Ecosystem** |  |  |  |  |  |
| Freshwater | 2.6 | 27.7 | 36.7 | 39.6 | 5191 |
| Terrestrial | 3.7 | 31.4 | 40.5 | 43.4 | 8365 |
| Marine | 2.3 | 30.5 | 40.8 | 43.7 | 2176 |
| **Habitat** |  |  |  |  |  |
| Forest | 4.5 | 33.9 | 43.7 | 46.8 | 5844 |
| Shrubland | 3.5 | 33.9 | 43.0 | 46.2 | 3225 |
| Coastal | 2.2 | 32.2 | 43.0 | 45.8 | 1901 |
| Savanna | 9.9 | 32.9 | 41.2 | 43.7 | 265 |
| Grassland | 3.3 | 31.3 | 39.9 | 42.7 | 3686 |
| Inland wetland | 2.6 | 27.9 | 36.9 | 39.7 | 5135 |
| Sea | 2.3 | 23.0 | 32.9 | 36.5 | 895 |
| Desert | 2.1 | 23.7 | 28.2 | 29.9 | 482 |
| **Region** |  |  |  |  |  |
| Africa | 10.4 | 38.9 | 42.9 | 44.0 | 1221 |
| Asia | 2.5 | 32.8 | 43.2 | 44.7 | 2279 |
| Australasia | 5.1 | 35.3 | 43.7 | 47.1 | 312 |
| Caribbean | 4.3 | 21.2 | 29.4 | 33.2 | 283 |
| Central America | 1.6 | 18.9 | 37.0 | 40.7 | 342 |
| Central Asia | 5.1 | 21.0 | 26.7 | 27.8 | 386 |
| Europe | 1.7 | 26.2 | 34.5 | 38.0 | 4580 |
| Middle East | 0.0 | 15.1 | 18.1 | 18.5 | 384 |
| North America | 6.2 | 21.3 | 26.1 | 26.1 | 327 |
| Oceania | 0.0 | 3.6 | 8.0 | 9.0 | 101 |
| South America | 0.8 | 25.6 | 33.9 | 37.8 | 753 |
| Developed | 2.4 | 29.8 | 38.8 | 42.5 | 4429 |
| Developing | 3.7 | 29.5 | 37.5 | 39.4 | 5226 |
| **Multilateral Environmental Agreement†** | | | |  |  |
| Ramsar | 2.5 | 29.5 | 38.5 | 40.9 | 4170 |
| AEWA | 2.3 | 25.4 | 33.7 | 36.3 | 4555 |
| ACAP | 17.0 | 41.6 | 45.9 | 45.9 | 47 |

† Ramsar = Ramsar Convention on Wetlands of International Importance. AEWA = African-Eurasian Waterbird Agreement under the Convention on Migratory Species (CMS); ACAP = Agreement on the Conservation of Albatrosses and Petrels under the CMS.
