# Supplementary material for: Protecting Important Sites for Biodiversity Contributes to Meeting Global Conservation Targets
Source: PLoS One. 2012 Mar 21;7(3):e32529. doi: 10.1371/journal.pone.0032529 (PMC3310057; doi:10.1371/journal.pone.0032529)
Supplement: Table S2 — Costs of IBA management. (DOCX) [file pone.0032529.s008.docx]

**Table S2.** Costs of IBA management.

|  | Country income level | | | |  |
| --- | --- | --- | --- | --- | --- |
|  | Low | Lower middle | Upper middle | High | Total |
| **All IBAs** |  |  |  |  |  |
| No. sites | 793 | 2,370 | 2,501 | 4,161 | 9,825 |
| Total area of IBAs (million ha^†^) | 127 | 245 | 359 | 174 | 906 |
| Mean management costs (thousand$/site) | 372 | 634 | 797 | 3,100 |  |
| Mean management costs ($/ha) | 152 | 57.8 | 50.9 | 910 |  |
| Median management costs ($/ha) | 4.89 | 25.3 | 19.1 | 307 |  |
| Minimum management costs ($/ha) | 0.14 | 0.31 | 0.07 | 2.24 |  |
| Maximum management costs (thousand $/ha) | 15.1 | 4.10 | 4.29 | 43.0 |  |
| Total management costs (million $) | 406 | 2,060 | 2,740 | 17,700 | 22,900 |
| **Currently protected IBAs** |  |  |  |  |  |
| No. sites | 435 | 1,126 | 1,058 | 2,692 | 5,311 |
| Total area of IBAs (million ha^†^) | 86.3 | 112 | 168 | 70.7 | 436 |
| Mean management costs (thousand$/site) | 390 | 884 | 691 | 2,370 | 1,080 |
| Mean management costs ($/ha) | 201 | 210 | 96.5 | 1,140 |  |
| Median management costs ($/ha) | 4.80 | 25.8 | 22.6 | 480 |  |
| Minimum management costs ($/ha) | 0.14 | 0.32 | 0.07 | 2.24 |  |
| Maximum management costs (thousand $/ha) | 17.2 | 31.2 | 3.36 | 46.3 |  |
| Total management costs (million $) | 235 | 1,380 | 1,020 | 8,870 | 11,500 |

^†^Adjusted to account for the 8.6% of sites with insufficient data
