# Supplementary material for: Protecting Important Sites for Biodiversity Contributes to Meeting Global Conservation Targets
Source: PLoS One. 2012 Mar 21;7(3):e32529. doi: 10.1371/journal.pone.0032529 (PMC3310057; doi:10.1371/journal.pone.0032529)
Supplement: Table S3 — List of countries excluded from the analysis of PA coverage of IBAs owing to incomplete data on IBAs and/or their PA coverage. (DOCX) [file pone.0032529.s009.docx]

**Table S3.** List of countries excluded from the analysis of PA coverage of IBAs owing to incomplete data on IBAs and/or their PA coverage.

American Samoa

Argentina

Chile

Cook Islands

French Guiana

Guyana

Kiribati

Kyrgyzstan

Nauru

New Caledonia

New Zealand

Niue

Papua New Guinea

Paraguay

Samoa

Solomon Islands

Tokelau

Tuvalu

USA

Vanuatu

Wallis and Futuna Islands
